# Association of Social Mobility With the Income-Related Longevity Gap in the United States: A Cross-Sectional, County-Level Study

**DOI:** 10.1001/jamainternmed.2019.6532

**Published:** 2020-01-21

**Authors:** Atheendar Venkataramani, Sebastian Daza, Ezekiel Emanuel

**Affiliations:** 1 Department of Medical Ethics and Health Policy, Perelman School of Medicine, University of Pennsylvania, Philadelphia; 2 Leonard Davis Institute of Health Economics, University of Pennsylvania, Philadelphia; 3 Center for Demography and Ecology, University of Wisconsin–Madison; 4 Department of Sociology, University of Wisconsin–Madison

## Abstract

**Importance:**

Despite substantial research, the drivers of the widening gap in life expectancy between rich and poor individuals in the United States—known as the *longevity gap*—remain unknown. The hypothesis of this study is that social mobility may play an important role in explaining the longevity gap.

**Objective:**

To assess whether social mobility is associated with income-related differences in life expectancy in the United States.

**Design, Setting, and Participants:**

This cross-sectional, ecological study used data from 1559 counties in the United States to assess the association of social mobility with average life expectancy at age 40 years by sex and income quartile among adult men and women over the period of January 2000 through December 2014. Bayesian generalized linear multilevel regression models were used to estimate the association, with adjustment for a range of socioeconomic, demographic, and health care system characteristics.

**Exposures:**

County-level social mobility, here operationalized as the association of the income rank of individuals born during the period of January 1980 through December 1982 (based on tax record data, averaged over the period January 2010 through December 2012) with the income ranks of their parents (averaged over the period January 1996 through December 2000) using the location where the parent first claimed the child as a dependent at age 15 years to identify counties.

**Main Outcomes and Measures:**

The main outcome was life expectancy at age 40 years by sex and income quartile.

**Results:**

The sample consisted of 1559 counties, which represented 93% of the US population in 2000. Each 1-SD increase in social mobility—equivalent to the difference between a low-mobility state, such as Alabama (ranked 49th on this measure), and a higher-mobility state, such as Massachusetts (ranked 23rd on this measure)—was associated with a 0.38-year (95% credible interval [CrI], 0.29-0.47) and a 0.29-year (95% CrI, 0.21-0.38) increase in county-level life expectancy among men and women, respectively, in the lowest income quartile. Estimates for life expectancies among county residents in the highest income quartile were smaller in magnitude and not robust to covariate adjustment (men: 0.10-year [95% CrI, −0.02 to 0.22] increase; women: 0.08-year [95% CrI, −0.05 to 0.20] increase). Increasing social mobility in all counties to the value of the highest social mobility county was associated with decreases in the life expectancy gap between the highest and lowest income quartiles by 1.4 (95% CrI, 0.7-2.1) years for men and 1.1 (95% CrI, 0.5-1.6) years for women nationally, representing a 20% decrease.

**Conclusions and Relevance:**

In this cross-sectional study, higher county-level social mobility was associated with smaller county-level gaps in life expectancy by income. These findings motivate further investigation of causal relationships between policies that shift social mobility and health outcomes.

## Key Points

**Question:**

Can social mobility—namely, differences in the ability of individuals to exceed the socioeconomic status of their parents—explain why gaps in life expectancy between rich and poor individuals in the United States are larger in some places than others?

**Findings:**

In this cross-sectional, ecological study of 1559 US counties, higher social mobility was significantly associated with higher life expectancy at age 40 years among men and women in the poorest income quartile and with smaller differences in life expectancy between the lowest and highest income quartiles.

**Meaning:**

Higher county-level social mobility was associated with smaller county-level longevity gaps between rich and poor individuals in the United States.

## Teaser

This cross-sectional study uses county-level data to assess the association of social mobility with income-related differences in life expectancy of individuals at age 40 years in the United States.

## Introduction

The strong association of higher socioeconomic status with longer life expectancy has been an enduring feature of population health for more than 2 centuries.^1,2^ The best-performing counties in the United States have average life expectancies that are now 20 years greater than the lowest-performing counties.^3^ Recent studies have demonstrated that the bulk of the longevity gap (the gap in life expectancy between rich and poor individuals) across counties is driven by the differences in life expectancies among the poorest residents in these counties.^4^ Since 2001, the differences in life expectancy at age 40 years between the richest and poorest quartiles of the income distribution grew from 8.6 years to 9.6 years among men and from 4.6 years to 5.7 years among women.^4^ These income-based longevity gaps are substantial, representing 25% of remaining life expectancy among men and 13% among women.^4^

These trends raise an urgent policy question regarding what intervention(s) might mitigate the longevity gap between high-income and low-income individuals living in the United States. Recent studies have suggested that neither access to medical care nor socioeconomic factors explain observed income disparities in longevity.^4,5^ Income inequality does not explain the longevity gap, either.^4^ Designing interventions to ameliorate the longevity gap requires reexamining its fundamental drivers.

We hypothesized that social mobility may play an important role in explaining income-related disparities in longevity. Social mobility reflects the ability of individuals to exceed the socioeconomic status of their parents. It is distinct from income inequality; areas with high levels of income inequality may have different rates of social mobility.^6,7,8^ Studies have demonstrated that social mobility in the United States varies greatly across space, with some US Census regions, such as the Northern Plains, affording high rates of mobility, whereas others, mainly in the South, afford little.^9^ A growing body of literature suggests that living in areas with low social mobility may harm individuals’ health by reducing their beliefs about future well-being, consequently increasing stress or diminishing the motivation to engage in healthy behaviors.^10,11,12,13,14,15,16,17^ The consequences of low area-level social mobility are likely largest for poorer individuals, for whom the potential for upward mobility is most salient.^6,8,18,19^

To our knowledge, no research has examined the association of area-level social mobility with differences in longevity by income. A large body of literature has examined the association of changes in individual-level socioeconomic status with health,^20,21,22,23^ but these associations do not necessarily reflect the contextual consequences of living in low-mobility areas.^24^ The few studies examining area-level social mobility have focused on associations with overall mortality rates.^10,11,17^

To address this gap in the literature, we conducted a cross-sectional, observational study using county-level data to assess the association of social mobility with life expectancy at age 40 years in the United States. We specifically examined whether the association varied by income, hypothesizing that associations would be strongest for individuals in the lowest income quartile and, consequently, that income-related life expectancy gaps would be smaller in higher-mobility counties.

## Methods

### Data Sources and Study Population

We used publicly available county-level data from the Health Inequality Project database (HIPD; https://healthinequality.org/) created by Chetty et al.^4,25^ The HIPD contains estimates of life expectancy at age 40 years. These data were derived from more than 1 billion tax records linked with US Social Security Administration data and are available for 1559 counties (representing 52% of US counties and 93% of the US population in 2000; rural counties were generally excluded because of small population sizes that limited calculation of life expectancies). For each county, the database includes life expectancy estimates for each population group defined by sex and income quartile for the period January 2000 through December 2014. Per University of Pennsylvania policy, institutional review board review was not required given the use of publicly available, preexisting, aggregate data. This study followed the Strengthening the Reporting of Observational Studies in Epidemiology (STROBE) reporting guideline.

The primary outcome of interest was the remaining years of life expectancy at age 40 years. The exposure of interest was social mobility, which was measured using HIPD data on relative income mobility.^19^ This measure, which is widely used in research on the causes and consequences of social mobility,^7,10,11,19,26^ represents the association of a child’s income rank in his or her birth cohort’s income distribution as an adult with the individual’s parents’ income rank in their income distribution. County-level measures of social mobility were previously unavailable until the work by Chetty et al,^19^ which used tax record data to estimate associations between incomes of individuals born from January 1980 through December 1982 at around age 30 years (ie, the average income over the period January 2010 through December 2012) and their parents’ income at the same age (ie, the average income over the period January 1996 through December 2000). Counties were assigned based on the location where the parent had claimed the child as a dependent at age 15 years.^19^ The social mobility measure ranged from 0 to 1, with larger values corresponding to lower social mobility (a value of 1 represents perfect dependence of the child’s income on the parents’ incomes). In the United States, the county with the highest social mobility had a value of 0.07, and the county with the lowest had a value of 0.66. To facilitate easier interpretation, we multiplied this measure by −1 so that higher values reflected greater mobility.

We obtained data on key covariates from the HIPD, including county-level measures of income inequality (the Gini coefficient) and average household income. These measures were used to adjust for economic characteristics that may be associated with social mobility.^6,19^ We also used HIPD data on unemployment rates (for 2000), residential segregation by income (for 2000), demographic information (percentage of black individuals and percentage of Hispanic individuals for 2000), percentage of uninsured individuals (for 2010), and per-capita health care expenditures (for the Medicare program for 2010). We chose these covariates because they have been well examined in the literature on longevity gaps.^4,27,28^

### Statistical Analysis

We first fitted local polynomial regressions to assess the unadjusted association of social mobility with life expectancy. Because of well-known differences in longevity gaps by sex, we conducted separate analyses for men and women. We separately estimated these regressions by income quartile to assess how associations varied across the income distribution, hypothesizing that the association between social mobility and longevity was largest for the lowest income quartile.

We then fitted a series of cross-sectional, Bayesian generalized linear hierarchical/multilevel regression models.^29^ Bayesian multilevel models are ideal for this research question because of the contextual nature of the exposure, their flexibility in allowing for dependence in life expectancy within relevant larger geographic areas (eg, counties within a given state are exposed to similar policy environments^30^), and their ability to more accurately predict outcomes under alternate scenarios.^31^

We first regressed life expectancy (by sex and income quartile) on the social mobility measure, which we standardized to facilitate easier interpretation of the regression coefficients. We first adjusted for logged and standardized average household income, standardized Gini coefficient for income inequality, and logged population. We then included additional economic, demographic, and health care access and spending variables to assess the sensitivity of the estimated associations to the inclusion of covariates. We specified state-specific random-effects models in all models to allow for the association of outcomes across counties within states, and 95% credible intervals (CrIs; the interval within which the true value of a parameter would fall at a probability of 95%) were estimated using weakly informative priors.^29,32^

The inclusion of covariates introduces a trade-off between adjustment for confounders vs capturing mechanisms underlying the causal chain linking social mobility to longevity. In the first case, estimated associations may be biased by failing to include key confounding variables. In the second case, including covariates could result in overadjustment.^11,33^ We tried to address possibilities by assessing the sensitivity of the models to including key covariates, while excluding from our main models measures that have been identified in the literature as potential consequences of changing social mobility, such as educational attainment or health,^10,34^ which are also likely to be associated with life expectancy. (We note that some potential moderators, such as education, may also be drivers of social mobility.^18^) However, in an additional analysis, we adjusted for level of education (specifically, the percentage of college graduates in the county) because assessing the remaining association between social mobility and health after adjusting for education may be useful in evaluating underlying mechanisms.

We then used coefficients from our main regression models to predict the change in the life expectancy gap for each county if social mobility in those counties were instead at the level of the best-performing county on this measure. We used these predictions to calculate the change in the nationwide life expectancy gap between the highest and lowest income quartiles associated with these large-scale improvements in social mobility.

All analyses were conducted using R version 3.5.1 (The R Foundation; replication data and code are available at https://github.com/sdaza/income-mobility-le-gap). Data analysis was conducted between January 2018 and September 2019.

### Sensitivity Analyses

We assessed the sensitivity of the results to several alternate model specifications. First, we used a robust regression method to assess the sensitivity of estimates to outlier observations.^35,36^ Second, we examined the different measures of social mobility. Specifically, we estimated models replacing our main social mobility variable with the average income rank of individuals born to parents in the lowest quartile of the income distribution (known as *absolute upward mobility*; higher values of this index reflect greater mobility). These data were calculated by Chetty et al^19^ and obtained from the HIPD. Third, we assessed the sensitivity of the estimates to adjustment for county-level in-migration and out-migration flows to account for potential bias from healthier individuals preferentially moving to high-opportunity areas.

## Results

### Descriptive Statistics

The study sample included all 1559 counties for which data on social mobility were available in the HIPD data set. Table 1 summarizes indicators of key characteristics for all counties in the sample as well as for counties in the lowest and highest quartiles of the social mobility measure. Compared with counties in the highest quartile, counties in the lowest quartile of social mobility had smaller mean (SD) population sizes (130 832 [324 502] residents vs 234 028 [608 657] residents), higher proportions of black residents (23.3% [16.3%] vs 2.8% [6.3%]), and lower proportions of Hispanic residents (3.7% [6.0%] vs 10.1% [16.7%]). Counties in the lowest quartile of social mobility also had lower mean (SD) levels of per-capita household income ($31 504 [$5071] vs $38 072 [$9300]) and greater income inequality (Gini coefficient, 0.45 [0.07] vs 0.38 [0.09]) as well as higher percentage of uninsured individuals (19.0% [3.8%] vs 15.6% [5.9%]) and higher levels of Medicare expenditures per capita ($9947 [$1254] vs $8524 [$1415]).

**Table 1.** Characteristics of Study Counties

Characteristic	Mean (SD)		
	Full Sample (N = 1559)	Social Mobility, Quartile	
		Lowest (n = 719)	Highest (n = 718)
Social mobility (relative income mobility, inverted)^a^	−0.27 (0.07)	−0.36 (0.03)	−0.18 (0.03)
Gini coefficient (for 2000)	0.40 (0.08)	0.45 (0.07)	0.38 (0.09)
Average household income (for 2000), $	34 855 (7578)	31 504 (5071)	38 072 (9300)
Population size (for 2000)	168 543 (399 949)	130 832 (324 502)	234 028 (608 657)
Black (for 2000), %	9.4 (13.1)	23.3 (16.3)	2.8 (6.3)
Hispanic (for 2000), %	6.5 (11.5)	3.7 (6.0)	10.1 (16.7)
Income segregation (for 2000)	0.04 (0.03)	0.04 (0.03)	0.05 (0.03)
Unemployed (for 2000), %	5.0 (1.6)	5.5 (1.5)	5.0 (2.1)
Uninsured (for 2010), %	17.2 (5.2)	19.0 (3.8)	15.6 (5.9)
Medicare expenses per capita (for 2010), $	9357 (1422)	9947 (1254)	8524 (1415)

^a^ Social mobility, ie, the relative mobility measure, was multiplied by −1 so that larger values reflect greater mobility. All data were obtained from the Health Inequality Project database.

### Unadjusted Analyses

The Figure displays the unadjusted, nonparametric associations of life expectancy at age 40 years with the social mobility measure by income quartile and sex. Longevity for both men and women was positively associated with relative income mobility. The magnitude of this association was greatest for men and women in the lowest quartile of the income distribution. Given this stronger association, a visual inspection revealed that the average life expectancy gap between the highest and lowest income quartiles decreased with greater county-level social mobility. The unadjusted gap in life expectancy between the poorest and richest income quartiles was 0.88 (95% CrI, 0.62-1.14) years larger for men and 0.25 (95% CrI, −0.01 to 0.51) years larger for women in counties in the lowest vs highest quartiles of social mobility.

**Figure.**
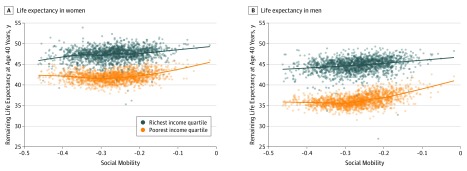
Unadjusted Estimates of the Association of Life Expectancy at Age 40 Years With Social Mobility by Income Quartile and Sex Estimates of the association of life expectancy at age 40 years with the social mobility measure obtained from separate nonparametric, local polynomial regression models estimated for women (A) and men (B) in the highest and lowest income quartiles. Each dot represents a county-income quartile observation, and the lines represent the fitted unadjusted, nonparametric association. Social mobility refers to the relative income mobility measure, which represents the association of a child’s income rank in his or her birth cohort’s income distribution as an adult with his or her parents’ income rank in their income distribution. This measure was multiplied by −1 so that higher values would reflect greater social mobility. A total of 1559 counties were included for all models.

### Adjusted Analyses

The association of county-level social mobility with longevity was confirmed in adjusted models. Table 2 provides estimates from models adjusting for county income, income inequality, and population and from models including these and additional covariates (full estimates including all covariates are provided in eTable 1 in the Supplement). In models adjusting for average income, income inequality, and county population size, each 1-SD increase in the social mobility measure—equivalent to the difference between a low-mobility state, such as Alabama, and a higher-mobility state, such as Massachusetts—was associated with an increase of 0.47 (95% CrI, 0.40-0.55) years in life expectancy at age 40 years for men in counties in the lowest income quartile. In models including additional covariates, the estimated increase in life expectancy associated with the same change in social mobility was 0.38 (95% CrI, 0.29-0.47) years. Estimates for women in the lowest income quartile were smaller in magnitude; after adjustment for average income, income inequality, and population size, each 1-SD increase in county-level social mobility was associated with a 0.34-year (95% CrI, 0.26-0.41) increase. After adjustment for additional covariates, the same 1-SD increase was associated with a 0.29-year (95% CrI, 0.21-0.38) increase in life expectancy.

**Table 2.** Adjusted Estimates of the Association of Life Expectancy at Age 40 Years With Social Mobility by Income Quartile and Sex^a^

Income Quartile	Adjusted Estimate (95% CrI)			
	Women		Men	
	Base Model^b^	Additional Covariates^c^	Base Model^b^	Additional Covariates^c^
1 (Poorest)	0.34 (0.26 to 0.41)	0.29 (0.21 to 0.38)	0.47 (0.40 to 0.55)	0.38 (0.29 to 0.47)
2	0.24 (0.16 to 0.31)	0.12 (0.03 to 0.21)	0.37 (0.29 to 0.45)	0.23 (0.14 to 0.32)
3	0.13 (0.05 to 0.22)	−0.01 (−0.11 to 0.09)	0.30 (0.21 to 0.38)	0.13 (0.04 to 0.22)
4 (Richest)	0.22 (0.12 to 0.32)	0.08 (−0.05 to 0.20)	0.18 (0.07 to 0.28)	0.10 (−0.02 to 0.22)

Abbreviation: CrI, credible interval.

^a^ Adjusted estimates were derived from Bayesian general linear multilevel models by sex and income quartile. Estimates reflect standardized coefficients, ie, the change in life expectancy at age 40 years associated with a 1-SD increase in the social mobility measure. Full estimates are provided in eTable 1 in the Supplement. All models include state-specific random effects to allow for the association of outcomes across counties within states. A total of 1559 counties were included for all models.

^b^ Base model regressions adjust for logged county-level average household income, *z* score of Gini coefficient, and logged total population size.

^c^ Additional covariate regressions adjust for logged county-level average household income, *z* score of Gini coefficient, logged total population size, logged percentage of black individuals, logged percentage of Hispanic individuals, logged unemployment rate, *z* score of percentage of uninsured individuals, and *z* score of percentage of Medicare expenditures per capita.

The estimated association of social mobility with life expectancy generally declined with increasing income quartiles. For the highest income quartile, each 1-SD increase in social mobility was associated with an increase in life expectancy at age 40 years of 0.22 (95% CrI, 0.12-0.32) years for women and 0.18 (95% CrI, 0.07-0.28) years for men in models adjusting only for income, income inequality, and population size. Estimates in models including additional covariates were smaller and no longer statistically significant (men: change in life expectancy, 0.10 years; 95% CrI, −0.02 to 0.22; women: change in life expectancy, 0.08 years; 95% CrI, −0.05 to 0.20).

Associations between social mobility and life expectancy were attenuated after adjusting for the share of college graduates. However, they remained substantively and statistically significant for the lowest income quartile (Table 3).

**Table 3.** Estimates of the Association of Life Expectancy at Age 40 Years With Social Mobility by Income Quartile and Sex, Adjusted for Area-Level Education^a^

Income Quartile	Adjusted Estimate (95% CrI)	
	Women	Men
1 (Poorest)	0.15 (0.05 to 0.25)	0.15 (0.07 to 0.24)
2	0.05 (−0.04 to 0.15)	0.11 (0.02 to 0.19)
3	−0.08 (−0.19 to 0.03)	0.02 (−0.07 to 0.11)
4 (Richest)	0 (−0.14 to 0.14)	−0.02 (−0.15 to 0.12)

Abbreviation: CrI, credible interval.

^a^ Models are identical to those presented in Table 2 in the Additional Covariates columns but are additionally adjusted for the county-level share of college graduates. Estimates reflect standardized coefficients, ie, the change in life expectancy at age 40 years associated with a 1-SD increase in the social mobility measure. A total of 1559 counties were included for all models.

Table 4 presents estimates of predicted gaps in life expectancy at age 40 years between the highest and lowest income quartiles associated with increasing social mobility in all counties to the same level as the best-performing county. For men, based on models including all covariates, life expectancy gaps were predicted to be smaller by 1.4 years (actual gap, 8.5 years; predicted gap, 7.1 years; difference, 1.4; 95% CrI of difference, 0.7-2.1), representing a 16.4% decrease. For women, the corresponding decline in the life expectancy gap was predicted to be 1.1 years smaller (actual gap, 5.5 years; predicted gap, 4.4 years; difference, 1.1; 95% CrI of difference, 0.5-1.6), a 20.0% decrease.

**Table 4.** Predicted Changes in Gaps in Life Expectancy at Age 40 Years Between Richest and Poorest Income Quartiles Associated With Counties Achieving the Highest Level of Social Mobility

Sex	Actual Gap, y	Base Model, Estimate (95% CrI), y		Additional Covariates, Estimate (95% CrI), y	
		Predicted Gap	Difference (Actual − Predicted Gap)	Predicted Gap	Difference (Actual − Predicted Gap)
Women	5.5	4.3 (3.5-5.1)	1.2 (0.6-1.9)	4.4 (3.3-5.6)	1.1 (0.5-1.6)
Men	8.5	6.8 (6.0-7.8)	1.7 (0.8-2.6)	7.1 (5.6-8.5)	1.4 (0.7-2.1)

Abbreviation: CrI, credible interval.

### Sensitivity Analyses

Results were similar in models accounting for undue influence of potential outliers (eTable 2 in the Supplement), and the substantive findings were unchanged when using an alternate measure of social mobility (eTable 3 in the Supplement). Estimates remained unchanged after adjustment for in-migration and out-migration rates (eTable 4 in the Supplement).

## Discussion

To our knowledge, this is the first study that assesses the association of area-level social mobility with income-related gaps in longevity. We found that for lower-income individuals living in the United States, higher county-level social mobility was associated with greater longevity and a lower longevity gap between these individuals and their richer counterparts. Across all counties, moving from the lowest to highest levels of social mobility was associated with a reduction in the longevity gap by 1.4 years among men and 1.1 years among women, approximately one-fifth of the longevity gap. Three points need emphasizing.

First, although the study design precludes making causal inferences, the findings suggest that recent declines in social mobility should be explored as a key contributor to the widening longevity gaps between high-income and low-income individuals living in the United States. The causes for these widening gaps have not been well elucidated, with empirical studies excluding many potential explanations, such as poverty rates, low educational attainment, employment rates, income inequality, segregation, and access to medical care.^4,28,37^ Conversely, the findings, if indeed reflective of a causal relationship, suggest that area-level social mobility may explain as much as 20% of the income-related longevity gap.

Second, the link between social mobility and the longevity gap may also be important in understanding emerging health trends within specific populations. For example, increasing mortality rates from alcohol, substance use disorder/substance use, and suicide among middle-aged individuals living in the United States have led to recent and stark divergences in health outcomes, including a reversal in life expectancy in some population groups.^38^ This trend has been linked to increasing despair from failing socioeconomic prospects^15,39,40,41^—an explanation consistent with the role of falling social mobility. Widening health gaps also appear to be associated with deindustrialization in certain geographic regions as well as rising rates of incarceration.^27,40,42,43^ Both deindustrialization and incarceration may have contributed to downward social mobility in the United States, particularly among low-income adults.^44,45^ Thus, declining social mobility may provide a more unifying explanation than widening income inequality for a variety of poor health trends, such as declines in life expectancy and the growing longevity gap.

Third, the association of area-level social mobility with the longevity gap suggests that policies to bolster social mobility can have important consequences for population health. Research elucidating the fundamental drivers of social mobility, which is critical to design effective public policy to address falling mobility, is ongoing.^18,19,45^ However, there is already evidence linking policies that shift social mobility with health outcomes. For example, adults randomized to receive vouchers to move to higher-income neighborhoods as part of the US Moving to Opportunity for Fair Housing program experienced some improvements in physical and mental health.^46^ By contrast, trade policies that led to the contraction of economic opportunities for manufacturing workers have been tied to rising mortality rates from drug overdose, suicide, and alcohol use.^40,42^

The evidence for interventions aimed earlier in the life course is more developed. Expansion of public health programs aimed at children and early-childhood interventions for children born into poverty may enhance both cognitive and noncognitive skills that raise prospects for upward mobility and improve health.^47^ Examples of successful programs that have achieved both objectives include Head Start, the Carolina Abecedarian Project,^48^ and the Perry Preschool program.^49^ Other early-life interventions, such as nurse-family partnerships^50^ and Medicaid expansions to young children,^51,52,53^ have also been shown to raise lifetime social mobility and improve health outcomes in adulthood.

### Limitations

This study has several limitations. First, despite advances in the measurement of social mobility, the county-level data we used were cross-sectional. Thus, the associations documented in this study cannot be interpreted as causal. Second, because we used aggregate data, the findings speak only to population averages and are subject to potential bias from ecological fallacy. In addition, the data were also aggregated over racial/ethnic groups, which precludes analyses of how the association of social mobility and life expectancy may vary across these dimensions. Third, the HIPD data only included information for metropolitan counties; it is possible that the association of social mobility with longevity differs in more rural areas. Fourth, the life expectancy data used in this study were estimated from tax records, which required extrapolation of mortality rates for older age groups and for race/ethnicity adjustment.^4^ Fifth, our area-level social mobility measure is retrospective and reflects county-level averages in actual or realized social mobility—that is, the association between incomes of parents and their children for a specific set of birth cohorts.^8^ The measure may not fully reflect area-level social mobility for the specific cohorts used to create the life expectancy measures or for future cohorts because the forces shaping economic opportunity may have changed over time.

The limitations of our analysis outline directions for future research. Studies that use experiments or natural experiments to focus on the consequences of policies or events that shift individual-level or area-level social mobility on health outcomes will be critical for assessing causality.^40,41,46,54,55,56^ Future studies should also seek to understand the drivers of these associations. For example, the association of social mobility with longevity likely reflects the consequences of the complex social, cultural, and political factors that shape social mobility in the first place.^8,19,45,57^ Understanding the relative importance of these factors may be critical in identifying interventions that promote both economic mobility and health. Similarly, examining key mechanisms underlying the association of social mobility with health—including biological processes, such as stress responses, and changes in economic expectations^13^—will also be important for developing effective interventions.

## Conclusions

We found that greater county-level social mobility was associated with smaller county-level longevity gaps by income in the United States. These findings motivate further investigation of causal relationships between policies that shift social mobility and health outcomes.

## Notes

Supplement. **eTable 1.** Full estimates of the adjusted association between county-level social mobility and county-level life expectancy at age 40 by income quartile (top versus bottom) and sex **eTable 2.** Estimates of the adjusted association between county-level social mobility and county-level life expectancy at age 40 by income quartile (top versus bottom) and sex using robust regression methods **eTable 3.** Estimates of the adjusted association between county-level social mobility and county-level life expectancy at age 40 by income quartile (top versus bottom) and sex using an alternate measure of social mobility **eTable 4.** Estimates of the adjusted association between county-level social mobility and county-level life expectancy at age 40 by income quartile (top versus bottom) and sex after additionally adjusting for county-level migration rates
