# Supplementary material for: Association of Social Mobility With the Income-Related Longevity Gap in the United States: A Cross-Sectional, County-Level Study
Source: JAMA Intern Med. 2020 Jan 21;180(3):429–36. doi: 10.1001/jamainternmed.2019.6532 (PMC6990844; doi:10.1001/jamainternmed.2019.6532)
Supplement: Supplement. — eTable 1. Full estimates of the adjusted association between county-level social mobility and county-level life expectancy at age 40 by income quartile (top versus bottom) and sex eTable 2. Estimates of the adjusted association between county-level social mobility and county-level life expectancy at age 40 by income quartile (top versus bottom) and sex using robust regression methods eTable 3. Estimates of the adjusted association between county-level social mobility and county-level life expectancy at age 40 by income quartile (top versus bottom) and sex using an alternate measure of social mobility eTable 4. Estimates of the adjusted association between county-level social mobility and county-level life expectancy at age 40 by income quartile (top versus bottom) and sex after additionally adjusting for county-level migration rates [file jamainternmed-180-429-s001.pdf]

Supplementary Online Content

Venkataramani A, Daza S, Emanuel E. Association of social mobility with the income-related longevity gap in the United States: a cross-sectional, county-level study. *JAMA Intern Med*. Published online January 21, 2020. doi:10.1001/jamainternmed.2019.6532

eTable 1. Full estimates of the adjusted association between county-level social mobility and county-level life expectancy at age 40 by income quartile (top versus bottom) and sex

eTable 2. Estimates of the adjusted association between county-level social mobility and county-level life expectancy at age 40 by income quartile (top versus bottom) and sex using robust regression methods

eTable 3. Estimates of the adjusted association between county-level social mobility and county-level life expectancy at age 40 by income quartile (top versus bottom) and sex using an alternate measure of social mobility

eTable 4. Estimates of the adjusted association between county-level social mobility and county-level life expectancy at age 40 by income quartile (top versus bottom) and sex after additionally adjusting for county-level migration rates

This supplementary material has been provided by the authors to give readers additional information about their work.

© 2020 Venkataramani A et al. *JAMA Intern Med.*

eTable 1. Full estimates of the adjusted association between county-level social mobility and county-level life expectancy at age 40 by income quartile (top versus bottom) and sex

	Women, Bottom Income Quartile	Women, Top Income Quartile	Men, Bottom Income Quartile	Men, Top Income Quartile
Relative income mobility (z-score)	0.29	0.08	0.38	0.1
	[0.21; 0.38]	[−0.05; 0.20]	[0.29; 0.47]	[−0.02; 0.22]
Log of average household income	0.97	0.21	1.64	1.2
	[0.57; 1.37]	[−0.37; 0.78]	[1.21; 2.06]	[0.61; 1.78]
Gini (z-score)	0.24	0.17	0.21	0.21
	[0.16; 0.32]	[0.06; 0.29]	[0.13; 0.28]	[0.10; 0.33]
Log of county population size	0.02	−0.08	−0.09	−0.11
	[−0.09; 0.12]	[−0.23; 0.07]	[−0.20; 0.02]	[−0.25; 0.04]
Income segregation (z-score)	−0.12	0.12	−0.02	0.2
	[−0.21; −0.02]	[−0.02; 0.27]	[−0.12; 0.08]	[0.06; 0.34]
Log of % Black American	−0.09	−0.07	−0.09	−0.04
	[−0.14; −0.03]	[−0.15; 0.01]	[−0.15; −0.03]	[−0.12; 0.04]
Log of % Hispanic	−0.22	−0.08	−0.06	−0.10
	[−0.30; −0.13]	[−0.19; 0.03]	[−0.15; 0.02]	[−0.21; 0.01]
Log of Unemployment Rate	0.42	−0.84	0.21	−0.69
	[0.14; 0.70]	[−1.21; −0.47]	[−0.08; 0.50]	[−1.06; −0.32]
% uninsured (z-score)	0.18	−0.12	0.42	−0.11
	[0.05; 0.30]	[−0.28; 0.03]	[0.28; 0.56]	[−0.26; 0.05]

© 2020 Venkataramani A et al. *JAMA Intern Med.*

Medicare expenses per capita (z-score)	−0.21	−0.31	−0.27	−0.39
	[−0.29; −0.12]	[−0.42; −0.20]	[−0.36; −0.19]	[−0.50; −0.28]
Random Effects - S.D. States	0.66	0.38	0.81	0.38
	[0.51; 0.83]	[0.25; 0.53]	[0.63; 1.02]	[0.22; 0.57]
N (counties)	1559	1559	1559	1559
N (states + DC)	51	51	51	51
Bayes R^2	0.34	0.16	0.5	0.25

Notes: Table reports full set of adjusted estimates, with 95% credibility intervals in brackets, by sex for each of the top and bottom income quartiles. Models are identical to those presented in **Table 2** of the main text (see **Table 2** notes for further details).

© 2020 Venkataramani A et al. *JAMA Intern Med.*

eTable 2. Estimates of the adjusted association between county-level social mobility and county-level life expectancy at age 40 by income quartile (top versus bottom) and sex using robust regression methods

	Women			Men	
**Income Quartile**	**Base model**	**Additional covariates**		**Base model**	**Additional covariates**
1 (Poorest)	0.35	0.30		0.47	0.37
	[0.27; 0.43]	[0.21; 0.39]		[0.39; 0.55]	[0.29; 0.46]
4 (Richest)	0.29	0.16		0.25	0.19
	[0.20; 0.38]	[0.05; 0.27]		[0.14; 0.35]	[0.07; 0.30]

Notes: Models are identical to those presented in **Table 2** of the main text, except here we use a Bayesian robust regression method using t-distributed errors to address any extreme outliers in the data (see Liang et al; 1992; *J.R. Stat. Soc B*; 54 (1): 3-40; Wang et al; *Bayesian Regression Modelling with INLA*, 2018). See **Table 2** notes for further details on covariates. N = 1,559 counties for each model.

© 2020 Venkataramani A et al. *JAMA Intern Med.*

eTable 3. Estimates of the adjusted association between county-level social mobility and county-level life expectancy at age 40 by income quartile (top versus bottom) and sex using an alternate measure of social mobility

	Women			Men	
**Income Quartile**	**Base model**	**Additional covariates**		**Base model**	**Additional covariates**
1 (Poorest)	0.41	0.37		0.43	0.32
	[0.32; 0.50]	[0.27; 0.48]		[0.34; 0.52]	[0.22; 0.43]
4 (Richest)	0.23	0.13		0.14	0.11
	[0.11; 0.35]	[−0.01; 0.28]		[0.02; 0.26]	[−0.02; 0.25]

Notes: Models are identical to those presented in **Table 2** of the main text except here we replaced our main social mobility variable with the average income rank of individuals born to parents in the bottom quartile of the income distribution (known as “absolute upward mobility” - higher values of this index reflect greater mobility). These data were calculated by Chetty et al (2014) and obtained from the HIPD. The coefficients can be interpreted in the same manner as in **Table 2** – i.e. the change in life expectancy at age 40 from each standard deviation change in the social mobility measure. See **Table 2** notes for full details on all covariates used in the model. N = 1,559 counties for each model.

© 2020 Venkataramani A et al. *JAMA Intern Med.*

eTable 4. Estimates of the adjusted association between county-level social mobility and county-level life expectancy at age 40 by income quartile (top versus bottom) and sex after additionally adjusting for county-level migration rates

Income Quartile	Women		Men
1 (Poorest)	0.29		0.38
	[0.20; 0.38]		[0.29; 0.48]
4 (Richest)	0.08		0.10
	[−0.05; 0.20]		[−0.03; 0.23]

Notes: Models are identical to those presented in **Table 2** of the main text, under the columns "Additional Covariates", except here we additionally adjusted for county in- and out-migration rates. The coefficients can be interpreted in the same manner as in **Table 2** – i.e. the change in life expectancy at age 40 from each standard deviation change in the social mobility measure. See **Table 2** notes for full details on other covariates used in the model. N = 1,559 counties for each model.

© 2020 Venkataramani A et al. *JAMA Intern Med.*
